# A comparison between healthcare workers and non-healthcare workers’ anxiety, depression and PTSD during the initial COVID -19 lockdown

**DOI:** 10.1016/j.puhip.2022.100267

**Published:** 2022-05-05

**Authors:** Inger Schou-Bredal, Tore Bonsaksen, Øivind Ekeberg, Laila Skogstad, Tine K. Grimholt, Trond Heir

**Affiliations:** a Faculty of Medicine, University of Oslo, Oslo, Norway; b Department of Health and Nursing Sciences, Faculty of Social and Health Sciences, Inland Norway University of Applied Sciences, Elverum, Norway; c Faculty of Health Studies, VID Specialized University, Sandnes, Norway; d Psychosomatic and CL psychiatry, Division of Mental Health and Addiction, Oslo University Hospital, Oslo, Norway; e Department of Research, Sunnaas Rehabilitation Hospital HF, Nesodden, Norway; f Department of Nursing and Health Promotion, Faculty of Health Sciences, Oslo Metropolitan University, Oslo, Norway; g Faculty of Health Studies, VID Specialized University, Oslo, Norway; h Department of Acute Medicine, Oslo University Hospital, Oslo, Norway; i Norwegian Center for Violence and Traumatic Stress Studies, Oslo, Norway; j Institute of Clinical Medicine, University of Oslo, Oslo, Norway

**Keywords:** Anxiety, Depression, COVID-19, Healthcare workers, Mental health, Pandemic, PTSD, Survey, COVID, the coronavirus, HCW, healthcare workers, PTSS, post-traumatic stress symptoms, CORONAPOP, the Population based cross-sectional survey on corona, NORPOP, the Norwegian Population Study, DSM-5, The Diagnostic and Statistical Manual of Mental Disorder. Fith EDition, PCL-5, Checklist for PTSD

## Abstract

**Objective:**

Several studies have found that Healthcare workers are vulnerable to mental health problems during the COVID-19 pandemic. However, few studies have made comparisons of healthcare workers (HCWs) and non-HCWs. The current study aimed to compare mental health problems among HCWs with non-HCWs during the initial lockdown of COVID 19.

**Study design:**

A population-based cross-sectional survey.

**Methods:**

The survey was conducted by means of an open web link between April and May 2020. Data were collected by self-report. The PTSD Checklist for DSM-5 (PCL-5) was used to assess posttraumatic stress.

**Results:**

A total of 4527 citizens answered the questionnaire and 32.1% were HCWs. The majority were female, under 60 years of age, and lived in urban areas. Among the HCWs, the majority were registered nurses working in hospitals. The prevalence were 12.8% vs 19.1% for anxiety, 8.5% vs 14.5% for depression and 13.6% vs 20.9% for PTSD among HCWs and non-HCWs respectively. The highest prevalence's for anxiety and PTSD among HCWs were found for those under 40 years of age and having low education level (<12 years).

**Conclusion:**

Mental health problems was significantly lower among HCWs compared to non-HCWs. However, the COVID-19 poses a challenge for HCWs, especially young HCWs and those with low level of education. Providing support, appropriate education, training, and authoritative information to the different members of the HCWs could be effective ways to minimize the psychological effect.

## 1 Introduction

Health professionals had to mobilize all their resources to provide emergency aid in a general climate of uncertainty during the outbreak of the coronavirus disease (COVID-19) pandemic. There were several challenges the healthcare workers (HCWs) had to face such as; dealing with critically ill contagious patients with an often unpredictable course of the disease, high mortality rates and lack of effective treatment or guidelines), disruption of normal support structures, the increased workload, fears of contagion for themselves and transmitting the disease to their families [[1], [2], [3], [4], [5]]. In addition, when resources were stretched to the limit, difficult decisions had to be made about who is suitable for invasive treatments such as life-support and who is not 2. Furthermore, due to the restrictions implemented because of the COVID-19, family and friends were not able to visit the patients, and staff may often feel guilt that the patient has “died alone” [6]. These challenges make healthcare workers (HCW) vulnerable to mental health problems including anxiety, depression, insomnia 7, and post-traumatic stress symptoms (PTSS) [8,9].

Reports of mental health problems among healthcare workers have persistently appeared during the current COVID-19 global health crisis. Results from systematic reviews and meta-analyses investigated the psychological impact on HCWs populations, found the pooled prevalence rate of anxiety ranged from 23.2% to 67.7%, and depression ranged from 12.1% to 55.9% [[10], [11], [12]]. Pappa et al. (2020) found that nursing staff exhibited higher prevalence estimates both for anxiety and depression than physicians. Vindegaard & Benros (2020) concluded from their systematic review that HCWs generally reported more anxiety, depression and sleep problems compared with the general population [13]. The outbreak of COVID-19, a life-threatening, and life-altering event, is considered traumatic enough to elicit PTSD. Previous studies related to the COVID-19 have found a prevalence rate of PTSD ranging from 4.7% to 16.7% [14,15], and post-traumatic stress symptoms (PTSS) ranging from 9.3% to 28.9% [14,16,17] among HCWs. It has also been reported that doctors report more PTSS than nurses [18].

The majority of COVID-19 studies investigating mental health problems have mainly focused on different health professionals or general populations or clinical samples. However, few studies were identified that included healthcare workers and non-healthcare workers in the same study. One study by Toh et al., 2021, found that healthcare workers reported better mental health than other essential workers and the general population [19].

The current study aimed to compare the prevalence of mental health problems (anxiety, depression, PTSD) among healthcare workers with non-healthcare workers during the initial lockdown of COVID 19 in Norway.

## 2 Methods

### 2.1 Design

A population-based cross-sectional survey (the CORONAPOP survey) was conducted by means of an open web link between April 8th, 2020 and May 20th, 2020. The web link was disseminated from Oslo University Hospital, Sunnaas Hospital, and the University of Oslo. In addition, the link to the survey was also disseminated on social media platforms, such as Facebook, Twitter, and Instagram by the individual researchers and other individuals who wanted to share the link to the survey and national and local newspapers. The study participants were Norwegian citizens aged 18 years or older. There were no exclusion criteria.

### 2.2 Measures

Sociodemographic (age, gender, education level, employment status before and during COVID-19 outbreak, living with spouse or partner, size of place of residence) and mental health data (anxiety, depression, and PTSD) were collected as self-report measures. Several measures identical to the ones used in the NORPOP health survey were employed in the study [[20], [21], [22], [23]]. Participants were asked if they were healthcare workers. The response options were: “no” and “yes”, If they answered yes, they were asked to indicate their profession (physiotherapist, doctor, registered nurse, auxiliary nurse, nursing assistant (care worker), social worker, cleaning staff, or other). If they answered “other”, they were given the option to specify their profession. In addition, they were asked to indicate where they worked (emergency unit, general practitioner office, health center, home nursing, nursing home, hospital, or other). If they answered “other” they were given the option to write where they worked.

### 2.3 Anxiety and depression

The questionnaire included the question: “Below is a list of health problems: Do you have, or have you had, any of these?” Among the listed problems were anxiety and depression. The answer options were: “no”, “yes”, and “last month” (i.e., during the COVID-19 lockdown). Those who confirmed having anxiety and/or depression, during the last month were classified as currently having a relevant mental health problem.

### 2.4 Posttraumatic stress symptoms

The PTSD Checklist for DSM-5 (PCL-5) was used to collect data on posttraumatic stress symptoms associated with the COVID-19 outbreak. To achieve a possible PTSD diagnosis, a respondent had to fulfil the DSM-5 symptom criteria for PTSD, except from the A criterion (having experienced accidental or violent death, threat of life, serious injury, or sexual violence) [24]. The 20-item PCL-5 is a self-administered questionnaire assessing the full domain of the DSM-5 PTSD diagnosis [25]. The instrument has four subscales, corresponding to each of the symptom clusters in the DSM-5. The symptoms endorsed were specifically linked to the COVID-19. Each item was scored on a 5-point scale (0 not at all, 1 a little, 2 moderately, 3 quite a bit, 4 extremely) to rate the extent to which the relevant symptom had bothered the person during the past month.

DSM-5 diagnostic guidelines [24] were applied to the PCL-5 to categorize participants as fulfilling PTSD symptom criteria or not. Participants indicating a score of 2 or above on at least one of five re-experiencing symptoms, one of two avoidance symptoms, two of seven symptoms of negative alterations in cognition and mood, and two of six arousal symptoms were classified as fulfilling the PTSD symptom criteria [[25], [26], [27]]. PCL-5 has good internal consistency, reliability, and validity [[26], [27], [28]]. The PCL-5 has been translated to Norwegian [29] and the original authors approved the final English back-translation. The Norwegian version of the PCL-5 has been validated and found to performed well as a diagnostic instrument for detecting PTSD [30].

### 2.5 Problems related to COVID-19

A questionnaire was developed to assess problems related to the COVID-19 pandemic.

The participants were asked if they had been infected with COVID-19, if they had been quarantined or isolated because of COVID-19 and whether or not they considered themselves to belong to the risk group for experiencing complication from COVID-19. They were also asked if they were generally worried about the pandemic. The answering obtains for these questions were yes or no.

### 2.6 Ethics

Ethical approval for conducting the study was given by the Regional Committee for Medical and Health Research Ethics (REC) (REC South East no. 130447). Informed consent was not necessary since the questionnaires were answered anonymously.

### 2.7 Statistics

IBM SPSS Statistics (version 24, IBM Corp., Armonk, NY) was used for statistical analyses.

Initial descriptive analyses employed frequencies and percentages. Cases with positive scores on the critical number of items in each symptom cluster were considered PTSD.

Chi-square tests were used to assess whether the frequency of anxiety, depression and PTSD, differed significantly between healthcare workers and non-healthcare workers. Previous research have found that gender, age, and level of education are associated with anxiety, depression and PTSD in the general population [[31], [32], [33]]. Thus, Chi-square tests were used to assess whether the frequency of anxiety, depression and PTSD differed significantly for gender, age and education level among healthcare workers and non-healthcare workers.

Employment status was dichotomized as working/in education versus not. Educational level was dichotomized as high (>12 years) versus low (<12 years). Age was dichotomized by participants age group <40 years of age and ≥40 years of age).

The significance level was 5%.

## 3 Results

A total of 4527 citizens completed the questionnaire. Approximately one-third of the respondents 32.1% (1453) were healthcare personnel. The majority in both groups, healthcare and non-healthcare workers were female, under 60 years of age, and lived in urban areas (Table 1).

**Table 1:** Demographics.

	Healthcare workers	Non healthcare workers	p value
	% (n)	% (n)	
**Gender**			<0.001
Female	89.3 (1294)	83.6 (2538)	
Male	10.7 (155)	16.4 (498)	
**Age group**			<0.001
<30	20.6 (299)	28.1 (856)	
30-39	25.1 (364)	28.0 (853)	
40-49	21.3 (310)	20.3 (619)	
50-59	23.2 (337)	13.9 (425)	
60-69	8.9 (129)	7.0 (214)	
70 or older	1.0 (14)	2.7 (83)	
**Place of Residence**			0.001
<2000 inhabitants	2.7 (39)	4.8 (147)	
2000–19.999 inhabitants	25.2 (366)	25.1 (765)	
20.000–99.999 inhabitants	22.4 (326)	25.0 (761)	
100.000 inhabitants or more	49.6 (720)	44.9 (1369)	
**Higher education level (> 12 years)**	84.3 (1225)	71.3 (2176)	
**Employed/in education/paid leave**			
Before lockdown	97.3 (1414)	83.7 (2552)	0.001
During lockdown	94.6 (1375)	74.8 (2282)	0.001
**Marital status**			0.001
Married	64.9 (943)	57.5 (1755)	
Boy/Girlfriend	6.5 (94)	7.3 (223)	
Widow	1.1 (16)	1.0 (32)	
Divorced	3.8 (55)	4.2 (128)	
Single	23.7 (345)	29.9 (912)	

*Note*. Statistical tests are Chi-Square tests. Missing data ranged from 0.00% to 0.64%.

The sample included many different types of healthcare workers, with the majority being registered nurses (34.9%), followed by physicians (5.7%) and physiotherapists (4.3%) (Table 2). Of the healthcare workers, 58.2% (1210) informed where they worked, with the majority working in hospitals, different kinds of institutions (i.e. psychiatry, geriatrics, elderly care, rehabilitation), or in primary health care (i.e. health centers, homes and environmental service, ambulance service, general practitioners office).

**Table 2:** Anxiety, depression and PTSD among different healthcare workers.

	N	%	Anxiety %	Depression %	PTSD %
**Total**	**1453**		**12.8**	**8.5**	**13.6**
Physiotherapist	62	4.3	6.5	6.5	8.1
Physician	83	5.7	6.0	4.8	11.0
Registered Nurse	507	34.9	12.2	7.3	11.8
Nurse assistant	93	6.4	16.1	12.1	20.9
Auxiliary nurse	61	4.3	24.6	13.1	26.7
Social worker	37	2.5	16.2	8.1	8.1
Other staff^a^	610	41.9	12.9	9.1	13.8

a Social educators, paramedics, milieu therapist, auxiliary personal without formal education.

More healthcare workers had been infected by the COVID-19 than non-healthcare workers, 2.0% versus 1.1%, p = 0.021, and had been in quarantine/isolation, 30.8% versus 26.9%, p = 0.007. However, significantly more of the healthcare workers were concerned about being infected compared to non-health workers, 15.2% versus 12.5%, p = 0.013. There was a significant difference between the groups with regard to social support, but the majority of both HCWs and non-HCWs had close friends or family who could provide social support if needed, 92.4% and 89.6%, p = 0.002 respectively.

As shown in Table 3 healthcare workers reported anxiety, depression, and PTSD less frequently than non-healthcare workers. No significant difference was found between physicians and nurses regarding anxiety (6.0% vs 12.2%, p = 0.09) and depression (4.8% vs 7.3%, p = 0.41).

**Table 3:** Mental health problems in healthcare and non-healthcare workers.

	Health personnel	Non-health personnel	P value
Anxiety	12.8%	19.1%	<.0001
Depression	8.5%	14.5%	<.0001
PTSD	13.6%	20.9%	<.0001

No significant difference was found for gender with regard to anxiety and depression in HCWs. In contrast, there was a significant gender difference among the non-HCWs with higher prevalence for anxiety and depression among females. The prevalence for PTSD symptoms was significantly higher for females in both groups.

Significantly more young adults (<40 years of age) reported anxiety in both groups. Regarding depression, there was no difference between younger and older (≥40 years of age) HCWs, but there was among the non-HCWs. The prevalence of depression was higher among older non-HCWs adults compared to younger non-HCWs adults (Table 4). The prevalence of PTSD symptoms was significantly higher for the younger age group in both groups.

**Table 4:** Anxiety, depression, and PTSD by age group, gender and education level.

	Healthcare Workers			Non-Healthcare Workers		
	Anxiety	Depression	PTSD	Anxiety	Depression	PTSD
	%	%	%	%	%	%
Female	13.1	8.1	14.3	20.7	15.2	22.2
Male	9.7	11.0	6.5	11.4	10.8	14.5
*p value*	*0.23*	*0.22*	*<0.01*	*<.001*	*.01*	*<0.001*

<40 years	18.1	10.0	16.1	23.5	16.1	23.3
>40 years	8.4	7.2	11.5	13.6	12.4	17.9
*p value*	<.001	.06	0.01	<.001	.004	<0.001

Education <12 years	20.9	12.3	21.8	26.2	19.2	28.1
Education >12 years	11.4	7.8	12.0	16.3	12.7	18.0
*p value*	<.001	.035	<.001	<0.001	<0.001	0.001

The majority (n = 1233) of HCWs had a higher level of education (>12 years). Those with lower education levels reported significantly more anxiety (20.9% vs 11.4%, p < 0.001), depression (12.3% vs 7.8%, p = 0.035). The prevalence of PTSD symptoms was significantly higher for the group with lower education levels (<12 years) in both HCWs and non-HCWs.

## 4 Discussion

This study investigated the prevalence of depression, anxiety, PTSD in a sample of Norwegian healthcare workers compared to non-healthcare workers during the COVID-19 outbreak. The prevalence rates for anxiety, depression, and PTSD of HCWs were lower than of non-HCWs. HCWs with a lower level of education (i.e. nursing assistance/auxiliary) reported more mental health problems than those with a higher level of education. Younger adults (<40 years of age) both among HCWs and non-HCWs reported more anxiety and PTSD.

Previous studies of coronavirus outbreaks, namely the Severe Acute Respiratory Syndrome (SARS) and the Middle East Respiratory Syndrome (MERS) epidemics have suggested that HCWs are at high risk for mental health problems [7,34]. A recent systematic review concluded that HCWs are more vulnerable to mental health problems due to the COVID-19 pandemic, because of the extreme exposure and high risk of infection due to their profession, as well as the lack of effective drugs and treatment strategies [13]. However, we found that the prevalence of anxiety, depression, and PTSD was significantly lower among HCWs compared to non-HCWs. Although the HCWs were confronted with difficult challenges and risks [2,4] they seemed to be mentally better prepared to handle the pandemic. One reason could be that the HCWs could better understand and classify COVID-19 related information than non-HCWs. Our findings are similar to two recent studies; Toh et al. (2021) conducted in Australia and Hummel et al. (2021) conducted in 8 European countries [35].

Although our results indicate that HCWs also are at risk of mental problems, our results showed a lower prevalence of mental health problems than reported by previous studies involving HCWs during the COVID-19 outbreak [[10], [11], [12],^35^]. This could be due to different measures used to assess mental health problems, or that healthcare systems vary greatly between countries or the great variation of infected cases needing hospitalization with or without ventilator treatment in the different countries. The incidence and mortality rate in Norway from COVID-19 during the outbreak was low compared to other countries (the EU's Centre for Disease Prevention and Control, March 2020). Thus, the health care system in Norway was not overwhelmed, and the infection rates and care for COVID-19 affected patients were under control. As a result, the HCWs in Norway could have been more confident about infection control and thus experienced lower levels of stress. This might explain why HCWs in Norway reported a more favorable mental health status than in other countries during the COVID-19 outbreak.

Previous studies of COVID-19 have found that nurses report more mental problems than doctors^.^[12, 36] This has been attributed to the fact that nurses are mostly female, spend more time on the hospital wards, provide direct care to COVID-19 patients, and are responsible for the collection of sputum for virus detection [2,16,37]. Thus, it is interesting to note that, in accordance with Guo et al. (2021) study, we also found no significant differences among female and male HCWs or nurses and physicians [38]. Both in Guo et al. study and ours, the HCWs were predominantly female and nurses, which is similar to the other studies on HCWs. Furthermore, rather than gender and profession, Guo et al. and we found that the education levels of the HCWs were associated with mental health problems. However, in contrast to Guo et al. (2021), we found that HCWs with a higher level of education reported less anxiety, depression, and PTSD. This could be explained by the fact that HCWs with a lower level of education more often lack medical training on COVID-19 and it has been shown that non-medically trained HCWs are at higher risk of adverse psychological outcomes, including PTSD, compared to medically-trained HCWs [15,39].

Younger age has been found a risk factor for anxiety and PTSD in previous COVID-19 studies of HCWs (see systematic review [40], and in the general populations [33,41]. Also, in the present study younger age among HCWs and non-HCWs appears to be associated with both anxiety and PTSD. As proposed by d’Etorre et al. (2021) the reason being that older HCWs are more experienced and often better equipped both professionally and psychologically to deal with the stress of the pandemic.

### 4.1 Limitations

There are some limitations that should be noted. Firstly, we used a cross-sectional design which limits the possibility to draw firm conclusions about causes and effects; therefore, caution should be taken in generalizing the findings. In addition, there was an undersampling of males, suggesting selection bias. The study was performed with an online questionnaire and people without internet and unable to use smartphones, tablets or computers could not be included in the study. Using single-item measures for anxiety and depression could by some be viewed as a limitation. However, single-item self-report measures have been shown to be reliable, as estimated by test-retest correlations [42] and correlations with clinical diagnosis [43].

## 5 Conclusion

The prevalence of mental health problems was significantly lower among HCWs compared to non-HCWs. Although the HCWs were confronted with difficult challenges and risks they seemed to be mentally better prepared to handle the pandemic than non-HCWs.

However, the COVID-19 also poses a challenge for healthcare workers. Especially young HCWs and those with low levels of education appeared to be more vulnerable for experience mental health problems among HCWs.

## Ethical approval

Ethical approval for conducting the study was given by the Regional Committee for Medical and Health Research Ethics (REC) (REC South East no. 130447). Informed consent was not necessary since the questionnaires were answered anonymously.

## Funding

This research did not receive any specific grant from funding agencies in the public, commercial, or not-for-profit sectors. Contents of this article are the sole responsibility of the authors.

## Author contributions

Conceptualization, T.K.G., T.B. I.S–B., L.S, Ø.E., and T.H.; methodology, T.K.G., T.B., I.S–B., L.S., Ø.E., and T.H.; validation, I.S–B., L.S., T.K.G., T.B., Ø.E., and T.H.; formal analysis, I.S–B., T.B., investigation, I.S–B., L.S, T.K.G., T.B., Ø.E., and T.H,; data curation, I.S–B.; writing – original draft preparation, I.S–B., writing – review and editing I.S–B., L.S, T.K.G., T.B., Ø.E., and T.H., visualization, I.S–B., project administration, T.K.G. All authors have read and agree to the published version of the manuscript.

## 6 Data sharing

The data presented in this study are available on request from the corresponding author by completion of the research project. The data are not publicly available due to ongoing publication from the project and different data protection regulations in the four involved countries.

## Declaration of competing interest

The authors declare that they have no known competing financial interests or personal relationships that could have appeared to influence the work reported in this paper.
